# In vitro quality control analysis after processing and during storage of feline packed red blood cells units

**DOI:** 10.1186/s12917-018-1458-4

**Published:** 2018-04-27

**Authors:** C. Blasi Brugué, Rui R. F. Ferreira, I. Mesa Sanchez, Rita M. C. Graça, Inês M. Cardoso, Augusto J. F. de Matos, Rafael Ruiz de Gopegui

**Affiliations:** 1 Department of Animal Medicine and Surgery, Veterinary Faculty, Autonomous University of Barcelona, 08193 Barcelona, Spain; 2 Department of Veterinary Clinics, Institute for Biomedical Sciences of Abel Salazar, University of Porto, 4050-343 Porto, Portugal; 3 Animal Blood Bank, 08023 Barcelona, Spain; 4 Animal Blood Bank, 4200-602 Porto, Portugal; 5 Animal Science and Study Centre, Food and Agrarian Sciences and Technologies Institute, University of Porto, Porto, Portugal

**Keywords:** Blood bank, Feline, Haemolysis, pRBC, Storage lesion, Transfusion

## Abstract

**Background:**

During the storage of packed red blood cells (pRBC), packed cell volume (PCV), bacterial contamination and percentage of haemolysis [percentage of free haemoglobin (HGB) in relation to the total HGB] are important quality parameters. Both PCV and haemolysis are indicators of the cellular integrity of stored units. There are no published experimental studies that evaluated these parameters during storage of feline pRBC using SAGM (adenine, dextrose, mannitol and sodium chloride) as the additive solution. The present study aims to (1) evaluate the quality of feline pRBCs stored in SAGM; (2) test for the semi-closed system’s suitability for use and risk of bacterial contamination; (3) establish the maximum storage time that may be appropriate to meet the criteria established by the United States Food and Drug Administration (US-FDA) guidelines for human blood banking; and (4) evaluate the need to calculate the percentage of haemolysis prior to the administration of units stored for more than 4 weeks.

Four hundred eighty nine feline pRBC units were analyzed. Bacterial culture, PCV and percentage of haemolysis were determined within 6 h after processing (t0). One hundred and eighty units were re-tested for haemolysis and PCV after 29–35 days of storage (t1) and 118 units after 36–42 days (t2).

**Results:**

Bacterial contamination was not detected in any pRBC unit. Mean PCV at t0 was 52.25% (SD: ±5.27) and decreased significantly (*p* < 0.001) during storage to 48.15% (SD: ±3.79) at t1 and to 49.34% (SD: ±4.45) at t2. Mean percentage of haemolysis at t0 was 0.07% (SD: ±0.06) and increased significantly (*p* < 0.001) to 0.69% (SD: ±0.40) at t1 and to 0.81% (SD: ±0.47) at t2. In addition, 13.88% and 19.49% of pRBC units exceeded 1% haemolysis at t1 and t2, respectively.

**Conclusions:**

According to the US-FDA guidelines for human blood banking that recommend a maximum of 1% haemolysis, the results of this study show that all feline pRBC units with less than 24 h of shelf life have low levels of haemolysis. However, units preserved up to 28 days can only be administered if tested for haemolysis before use, since 13.88% units exceeded the 1% limit. The semi-closed system was considered safe for use as bacterial contamination was not detected in any pRBC unit.

## Background

In the last decades, feline transfusion medicine has significantly evolved, the use of packed red blood cells (pRBC) rather than whole blood was described in 15% and 47% of feline patients submitted to transfusion [1, 2].

Guaranteeing the safety of haemocomponents is essential and must be of the utmost importance for blood banks. Reducing the risks of transfusion reaction requires ensuring that the product is free of blood-borne pathogens; that there is absence of bacterial contamination; and that erythrocyte antigens are determined to avoid allogeneic immune reactions. Furthermore, the viability of erythrocytes must also be guaranteed.

Reported recommendations for storage of feline pRBC using additive solutions and citrate-based anticoagulants vary between 30 and 42 days at 2–6 °C [3–5]. However, there are no experimental reports aiming to evaluate haemolysis or bacterial growth in feline pRBC units stored in such conditions. There is just one publication that tested for haemolysis and bacterial growth in 27 feline fresh WB units, using an open collection system [6].

During storage, blood cells maintain their metabolic activity, releasing byproducts to the media and suffering from biologic and immunologic changes, which may affect red blood cells (RBCs) function and survival. These changes are known as storage lesions, and may cause adverse effects on the recipients [4, 7–11]. Haemolysis, as one result from these processes, may be regarded as an indicator of storage lesions. Haemolysis percentage is considered the standard in human medicine to determine pRBC’s shelf life [12].

Released byproducts, mainly produced by leucocytes and platelets, contribute to RBC haemolysis and are an important cause for transfusion reactions; most of them of the febrile non-haemolytic type [13, 14].

Units’ haemolysis is highly influenced by the availability of ATP, produced mainly via anaerobic glycolysis through the Embden-Meyerhof pathway, catalyzed by phosphofructokinase (PFK). ATP is essential to maintain erythrocyte function and stability [15–17]. During storage, hydrogen ion activity inhibits PFK, and it increases with time as lactic acid accumulates due to anaerobic glycolysis [18, 19]. As the lack of energy sources becomes critical, the RBC’s metabolic activity fails to maintain normal functionality, its membrane elasticity reduces, intracellular viscosity increases, and morphological changes occur, resulting in haemolysis [4, 7, 20–25].

Furthermore, these changes also affect the RBCs capacity of oxygen distribution and CO_2_ removal from tissues [7]. Once in circulation, transfused RBCs either reassume the original biconcave shape within 24 h or they will be removed by the reticuloendothelial system, thus reducing their survival time on the recipient [26–28].

By adding saline, dextrose, adenine, and mannitol to the additive solution used to store RBCs, it is possible to delay the loss of ATP and increase the pRBC lifetime up to 44 days [29, 30].

Mechanical and environmental factors may also affect the RBCs viability [28, 31]. Haemolysis is highly influenced by the processing, storage and administration protocols, including the delay between collection and separation, centrifugation speeds, sterility of the units, intravenous tubing gauge, occluded needles, storage temperature, and the units’ PCV [31–35].

Therefore, it is important that such factors are optimized, standardized and monitored, and that quality controls are periodically done to ensure that the units are not damaged and the procedures are safe and effective [32].

It is important to ensure that the transfused units are minimally haemolyzed, not only to ensure that the transfused RBCs are functional, but also because free haemoglobin, resulting from haemolysis, may be an important cause of transfusion reaction, mainly of the nonimmune-mediated haemolytic type [36]. Acute fatal or life-threatening transfusion reactions associated to the administration of haemolysed pRBCs in dogs have been described, with clinical signs similar to an acute haemolytic reaction [10]. When free HGB surpasses plasma and cellular binding capacities, it acts as an important vasoactive and redox active protein [37, 38]. It is also important to notice that free HGB is potentially toxic for the vascular, myocardial and renal systems, the toxicity depending on exposure time and concomitant diseases such as renal insufficiency [37, 39–46].

PCV reduction, bacterial contamination, and haemolysis are important pRBC quality parameters that allow for addressing the cellular integrity of stored units, and are limiting factors for the shelf-life of stored red blood cells [47]. The Council of Europe and the U.S. Food and Drug Administration (US FDA) guidelines for human blood banking recommend that, at the end of storage, no more than 0.8% and 1% haemolysis, respectively, is surpassed to ensure that no haemolysed units are transfused to patients [47, 48]. Similar recommendations, however, lack in veterinary medicine.

The present study aimed to (1) evaluate the quality of feline pRBCs stored in SAGM; (2) test for the semi-closed system’s suitability for use and risk of bacterial contamination; (3) establish which maximum storage time may be appropriate to meet the criteria established by the United States Food and Drug Administration (US-FDA) guidelines for human blood blanking; and (4) evaluate the need for quality control analysis before administration of units stored for more than 4 weeks.

## Methods

From all units collected between 2014 and 2016 at the Animal Blood Bank in Spain and Portugal (Banco de Sangre Animal, Barcelona, Spain\ Banco de Sangue Animal, Porto, Portugal), one out of each 5 units was randomly selected for quality control analysis. Thus, a total of 489 feline fresh whole blood (FWB) units were analyzed. All donors were indoor healthy cats weighing 4–9 Kg that had been vaccinated, dewormed, tested for Feline Immunodeficiency Virus, Feline Leukemia virus (Uranotest FeLV-FIV, Uranovet, El Prat de Llobregat, Barcelona), *Mycoplasma haemofelis, Candidatus Mycoplasma haemominutum and Candidatus Mycoplasma turicensis* (PCR analysis by Genevet, Algés, Portugal). Complete blood counts and chemistry profiles prior to the collection procedures were within normal reference ranges. No animals where directly involved in this study, all the data was obtained from the routinary procedures performed at the animal blood bank, no unnecessary procedures were done to blood donors. All blood samples were collected after signed informed owner consent. This study was conducted according to European legislation (86/609/EU).

Whole blood units were collected using a specific feline semi-closed system without leukocyte depletion filters, consisting of a 50 ml syringe and a primary blood bag collection attached to the syringe with a sterile connector (CompoDock, Fresenius SE, Hesse, Germany). The collection system was sealed, sterilized with Ethylene Oxide (EtO), and 8 mL of CPD (tri-sodium citrate, sodium phosphate and dextrose) were added as anticoagulant to the syringe, under sterile conditions using a laminar flow hood (Cruma FL-1, Diantech Solutions S.L., Barcelona, Spain).

After a complete physical examination, an intravenous catheter was placed on the cephalic vein, and mild sedation was applied intravenously using ketamine and diazepam. The use of NMDA receptor antagonist and benzodiazepine combination is commonly reported in the bibliography [49]. A combination of tiletamine and zolazepam has been reported safe for feline blood donation [50]. Once sedated, donors were placed in sternal recumbency, and the puncture area over the jugular vein was shaved and aseptically prepared using chlorhexidine and alcohol. Jugular venipuncture was performed and blood was withdrawn applying negative pressure by gently pulling manually the syringe plunger. A maximum of 10–12 ml/kg was collected [49]. During collection, the syringe was gently stirred to allow proper contact of the blood with the anticoagulant. The collected blood was then transferred to the blood bag through the sterile connection ensuring the maintenance of a closed environment. After that, the tubing was sealed (Composeal, Fresenius Kabi, Hesse, Germany), units were stored at room temperature (22 ± 2 °C) and processed within 24 h. The volume of pRBC units was calculated on the basis of their weight, considering that 1 mL of pRBC weights 1.085 g [51].

Units were gently mixed and placed in the centrifuge cups (Megafuge 40R, Thermo Scientific, Massachusetts, USA) eliminating void space by using manufactured plastic adaptors. Weight differences under 0.3 g between opposite cups were tolerated. Whole blood units were centrifuged at 2000 g for 15 min at 20 °C (64.4 °F), with 80 s of acceleration and 110 s of deceleration.

Plasma was then expressed into a secondary transfer bag using a sterile connection of polyvinyl chloride tubing (CompoDock, Fresenius SE, Hesse, Germany), and 10 mL of SAGM (adenine, dextrose, mannitol and sodium chloride) were added to the pRBC unit under a laminar flow hood (Cruma FL-1**,** Diantech Solutions S.L., Barcelona, Spain).

For sampling purposes, pRBC units were gently mixed by inversion, and a 3 mL aliquot was aseptically collected using a sterile connection with a sample bag (Macopharma, Mouvaux, France), and analyzed (*t* = 0) for PCV, total HGB and supernatant HGB.

PCV was measured by microhaematocrit centrifugation [52]. Total HGB was measured using a specific analyzer (Hb 201 System, HemoCue Inc., California, USA), according to the manufacturer’s protocol. After centrifugation (Centrifuge IEC Centra CL3R, Thermo Scientific, Massachusetts, USA), supernatant HGB was determined by spectrophotometry using an analyzer for low values of HGB (Plasma Low Hb, HemoCue Inc., California, USA), according to the manufacturer’s protocol. The percentage of haemolysis was obtained using the following formula [32]:

*% haemolysis = Supernatant HGB (g/L) x (100-PCV) / Total HGB (g/L).*

Bacterial cultures were performed in all units at t0 by adding, under sterile conditions, 2,5 mL of pRBC to aerobic culture bottles with specific growth medium (Bact/Alert PF, Biomerieux, Marcy l’Etoile, France), followed by incubation at 37 °C (98.2 °F) and continuous examination for 14 days using a specific analyzer (Bact/Alert 3D, Biomerieux, Marcy l’Etoile, France).

Packed RBCs units were stored at 4 °C in a dedicated refrigerator (Medika 250, Fiocchetti, Luzzara, Italy). One hundred and ninety-one units were used for clinical purposes, and 298 units were retested for haemolysis and PCV after storage.

Data was grouped, according to storage times, in 3 groups; group 1 evaluated within 6 h after processing (t0); group 2 re-tested after 29–35 days of storage (t1); group 3 re-tested after 36–42 days of storage (t2).

Results were analyzed with statistical software (SPSS, version 22.0.0, IBM, Illinois, USA). Normal distribution of data was assessed with the Kolmogorov-Smirnov test. ANOVA F de Snédècor and Tukey’s post hoc Test were used to assess for haemolysis, PCV, or total HGB differences between evaluation moments. The relationship between the number of units that exceeded 1% of haemolysis and the storage duration was assessed using Chi Square. Values were considered significant at *p* < 0.001.

## Results

A total of 489 pRBC units where tested for bacterial contamination, PCV and haemolysis at t0, 180 were retested for PCV and haemolysis after 29–35 days of storage (t1) and 118 units tested for these same parameters after 36–42 days of storage (t2). The remaining units were used before retesting.

Data for all variables were normally distributed. Haemolysis, PCV and total HGB values from pRBC units are displayed in Table 1. There were significant PCV and haemolysis differences between the evaluation moments (*p* < 0.001) (Table 1).

**Table 1:** PCV, Total Haemoglobin and haemolysis values at t0, t1 and t2: t0: units evaluated within 6 h after processing; t1: units re-tested after 29–35 days of storage; t2: units re-tested after 36–42 days of storage

	t0		t1		t2		F	*p*
	M	SD	M	SD	M	SD		
PCV (%)	52.25	5.27	48.15	3.79	49.34	4.45	55.078	<.001
Total Haemoglobin	11.15	8.49	10.13	7.87	10.24	8.04	2.069	.127
Haemolysis (%)	.07	.06	.69	.40	.81	.47	578.540	<.001

*M* mean value, *SD* standard deviation, *PCV* Packed cell volume, *F* F-value

Significant value *p* < 0.001

The mean percentage of haemolysis at t0 was 0.07%, (SD: ±0.06), with all units below the maximum of 1% allowed by the US FDA, and the mean PCV was 52.2% (SD: ±5.27). At t1, the mean percentage of haemolysis was 0.69% (SD: ±0.4), with 25 (13.88%) units surpassing 1% haemolysis, and the mean PCV was 48.15% (SD: ±3.79). At t2, the mean percentage of haemolysis was 0.81% (SD: ±0.47), with 23 (19.49%) units presenting more than 1% haemolysis, and the mean PCV was 49.34% (SD: ±4.45) (Table 2).

**Table 2:** Differences between t0, t1 and t2: t0: units evaluated within 6 h after processing; t1: units re-tested after 29–35 days of storage; t2: units re-tested after 36–42 days of storage

	Group	Dif	SE	*p*
PCV (%)	t0-t1	4.107	.419	<.001
	t0-t2	2.915	.492	<.001
Haemolysis (%)	t0-t1	−.620	.023	<.001
	t0-t2	−.743	.028	<.001
	t1-t2	−.123	.032	<.001

*Dif* differences between mean values of each group, *SE* standard error, *PCV* Packed cell volume

Significant value *p* < 0.001

**Fig. 1:**
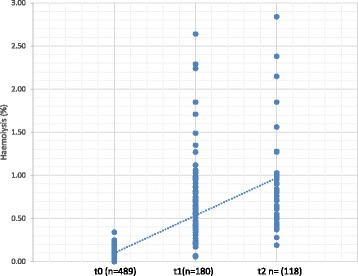
Progression of haemolysis during storage of feline pRBC units t0: units evaluated within 6 h after processing; t1: units re-tested after 29–35 days of storage; t2: units re-tested after 36–42 days of storage

**Fig. 2:**
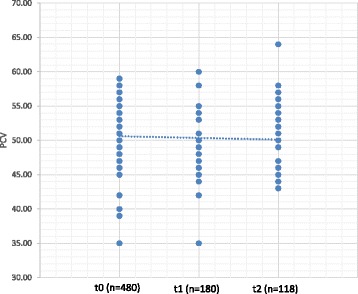
Progression of PCV of feline pRBC units during storage. PCV: Packed cell volume; t0: units evaluated within 6 h after processing; t1: units re-tested after 29–35 days of storage; t2: units re-tested after 36–42 days of storage

Results show that PCV decreased between t0 and t1, and between t0 and t2, while haemolysis increased between t0 and t1, between t0 and t2 and between t1 and t2. There was no statistical difference in PCV between t1 and t2 (Figs. 1 and 2 and Table 3). There was a statistically significant relation between the evaluation moments and the number of units exceeding 1% of haemolysis (χ^2^ (2) = 46.694; *p* < 0.001).

**Table 3:** Number of packed red blood cells (pRBC) units exceeding 1% of haemolysis at t0, t1 and t2

	≤ 1% (*n* = 739)	> 1% (*n* = 48)
t0 (*n* = 489)	100% (*n* = 489)	0%
t1 (*n* = 180)	86.12% (*n* = 155)	13.88% (*n* = 25)
t2 (*n* = 118)	80.51% (*n* = 95)	19.49 (*n* = 23)

χ^2^ (2) = 87.778; *p* < .001

t0: units evaluated within 6 h after processing; t1: units re-tested after 29–35 days of storage; t2: units re-tested after 36–42 days of storage; ≤ 1%: units not exceeding 1% of haemolysis; > 1%: units exceeding 1% of haemolysis; n: number of units

## Discussion

Percentage of haemolysis is the mostly used parameter to establish the viability of pRBC manufacturing and conservation protocols [12]. It is influenced by blood cells metabolic activity and release of substances into the supernatant, but also by environmental factors such as temperature, or collection and processing protocols. As an indicator of storage-related cell damage, it is also an important parameter for the transfusion safety. The United States Food and Drug Administration guidelines for human blood banking recommend a maximum of 1% haemolysis to ensure that no haemolytic products are transfused to patients, and use haemolysis as one of the main parameters to approve additive solutions and conservation protocols [53, 54].

Only two recent studies aimed to evaluate storage lesions in feline pRBCs [11, 12]. Significant supernatant increases of lactate, ammonia, sodium and chloride, and decreases of glucose and potassium levels were described.

To the authors’ knowledge, there are no previous publications on the quality control of feline pRBC that reported their haemolysis after collection and during storage with SAGM or other preservative solutions. Similar to studies performed in human and canine pRBC units, there was an increase in the haemolysis levels over storage time. We can assume that this might be due to the progressive depletion of ATP and the effect of the proinflammatory substances produced mainly by leucocytes and platelets [4, 16, 55, 56]. Our results indicate that after 29–35 days of storage, nearly 14% of the units suffered more than 1% haemolysis, and such value increased to almost 20% after 36–42 days. Hence, it could be postulated that units preserved for more than 28 days should only be administered once tested for haemolysis to ensure that its value does not compromise safety and efficacy of the transfusion.

In one previous study, 164 human units were analyzed after 42 days of storage in a hospital-based transfusion service, and 13.4% exceeded 0.8% of haemolysis [55]. In our series, haemolyzed units after similar storage times were more common, possibly due to higher difficulties in the collection processes associated to the need for negative pressure, smaller vein diameters, and a shorter lifespan of feline pRBC (77 days) [57].

Previous studies of our group in dogs concluded that 6% of the units surpassed 1% haemolysis at 35 days, although this number increased to 51% at 42 days of storage [58]. Interestingly, feline pRBCs showed a higher proportion of haemolysed units at fifth week of storage, but much lower than canine at sixth week (18.57% vs 51%). The explanation for such difference may reside in the distinct RBC metabolism between species or be due to differences in the feline blood bags that, being smaller and with a higher surface-to-volume ratio, allow for a higher capacity for gas exchange and faster temperature homogenization.

In blood banks and clinical practices, pRBC units should always be checked for visible alterations indicating haemolysis before its use. Although clinically useful, it is considered a non-reliable and subjective method, as it often overestimate the haemolytic status of the pRBC units since even as little as 0.09% of haemolysis causes the appearance of an evident pink discoloration of the supernatant [53, 54]. HaemoCue has been compared to the gold standard tetramethylbenzidine spectrophotometric method and reported to be a reliable objective method to measure plasma HGB for routine quality control and validation process, being a faster, easier and reliable system [54].

In our series, mean PCV was maintained during storage, with a slight decrease over time, from 52.2% (SD: ±5.27) at t0 to 48.15% (SD: ±3.79) at t1, and to 49.34% (SD: ±4.45) at t2. These results contradict those previously reported in canine or human pRBC. Canine and human pRBC PCV increased during storage, a phenomenon explained by the influx of water into RBCs caused by cell membrane damage during storage and the osmotic effects of the supplementary solutions [56, 58].

The differences between other mammalian and cats may be attributed to physiologic and metabolic particularities of the latter that may lead to different morphologic changes during storage. One other possible cause for these differences could be the smaller size and surface to volume ratio of the feline pRBC that may result in a lower RBC osmotic fragility and consequently a reduced capability of swelling, when compared to canine RBCs, leading to a membrane destruction earlier in the swelling process, thus not allowing for PCV to increase [59]. These hypotheses warrant future studies on the morphologic changes of feline erythrocytes during storage.

Closed collection systems are not always available in feline transfusion medicine. The alternatives are semi-closed or open systems, which are used in many blood banks and veterinary hospitals, but the latter preclude storage due to the high risk of bacterial contamination, leading some blood banks to use semi-closed collection systems [49]. The semi-closed system, used in this study, was considered safe since no bacterial contamination was detected in any pRBC unit and haemolysis (mean 0.07%, SD ±0.06) was under 1% in all units before storage, similar to the results of canine collections using close systems (0.09%, SD ±0.06) [58].

To the authors’ knowledge there are no previous reports aiming to validate the semi-closed collection systems for use in blood banking. In previous studies, contamination by *Serratia* spp. and *Pseudomona* spp. have been described in feline WB and pRBC units using open collection systems, and one pRBC unit collected with a semi-closed system was tested positive to *Pseudomonas fluorescens* after color changes were noted [60, 61]. Moreover, two studies performed blood cultures in feline pRBC collected with open systems, testing 10 feline pRBC at day 32 of storage in one study and 6 units at day 42 of storage on the other. Both studies reported negative blood cultures at the end of storage [12, 62].

One limitation of this study was that blood culture was performed only 24 h after collection, and not repeated after the storage period, although contamination during storage is considered unlikely, sensitivity for bacterial contamination during processing might be higher after a long storage period. However, no units showed signs of bacterial growth (e.g. dark purple to black or green discoloration) or visible signs of clotting or fibrin in the blood bag at any time during storage, although the absence of these indicators does not preclude de possibility of bacterial contamination [61, 63].

Another limitation was that units were not re-tested for haemolysis before 29 days, which was because feline pRBC are a valuable resource, and the design of the study was thought to optimize the availability of the pRBC for clinical use.

Other quality parameters that were not analyzed in our study but might have been necessary for a complete quality control analysis include, as routinely performed in human medicine, biochemical measurements and RBC morphology analysis, including red cell shape, size, cell surface markers, glucose utilization rates, lactate production rates, and ATP levels and utilization rates. Our aim, by analyzing the storage times and processing methods in regard to haemolysis and PCV, was to describe representative indicators for red blood cell storage damage.

## Conclusions

Considering the U.S. FDA guidelines for human blood banking that recommend a maximum of 1% haemolysis to ensure that no haemolytic products are transfused to patients, our results evidenced that all pRBC units with less than 24 h of shelf life have negligible haemolysis. However, units preserved for more than 28 days can only be safely administered if tested for haemolysis before its use, since 13.88% units exceeded the 1% limit at 35 days of storage. Furthermore, our results indicate that the semi-closed collection systems, when manipulated in sterile conditions, are reliable for feline blood banking. Further studies are needed to assess storage lesions and erythrocyte morphologic changes in feline RBCs during storage of pRBC.

## Abbreviations

ATP: Adenosine triphosphate
CPD: Tri-sodium citrate, sodium phosphate and dextrose
FWB: Fresh whole blood
HGB: Haemoglobin
PCV: Packed cell volume
PFK: Phosphofructokinase
pRBC: Packed red blood cells
RBC: Red blood cells
SAGM: Additive solution (adenine, dextrose, mannitol and sodium chloride)
SD: Standard deviation
US FDA: United States Food and Drug Administration
WB: Whole blood

## References

[1] Castellanos I, Couto CG, Gray TL (2004). Clinical use of blood products in cats: a retrospective study (1997-2000). J Vet Intern Med.

[2] Klaser DA, Reine NJ, Hohenhaus AE (2005). Red blood cell transfusions in cats: 126 cases (1999). J Am Vet Med Assoc.

[3] Jagodich TA, Holowaychuk MK (2016). Transfusion practice in dogs and cats: an internet-based survey. J Vet Emerg Crit Care (San Antonio)..

[4] Obrador R, Musulin S, Hansen B (2015). Red blood cell storage lesion. J Vet Emerg Crit Care.

[5] Gibson G, Abrams-Ogg A, Day MJ, Kohn B (2012). Canine transfusion medicine. Canine and feline haematology and transfusion medicine.

[6] Spada E, Proverbio D, Baggiani L, Bagnagatti De Giorgi G, Ferro E, Perego R (2017). Change in haematological and selected biochemical parameters measured in feline blood donors and feline whole blood donated units. J Feline Med Surg.

[7] Barshtein G, Manny N, Yedgar S (2011). Circulatory risk in the transfusion of red blood cells with impaired flow properties induced by storage. Transfus Med Rev.

[8] Kor DJ, Van Buskirk CM, Gajic O (2009). Red blood cell storage lesion. Bosn J Basic Med Sci.

[9] Roback JD, Neuman RB, Quyyumi A, Sutliff R (2011). Insufficient nitric oxide bioavailability: a hypothesis to explain adverse effects of red blood cell transfusion. Transfusion.

[10] Patterson J, Rousseau A, Kessler RJ, Giger U (2011). In vitro lysis and acute transfusion reactions with hemolysis caused by inappropriate storage of canine red blood cell products. J Vet Intern Med.

[11] Cummings KA, Abelson AL, Rozanski EA, Sharp CR (2016). The effect of storage on ammonia, cytokine, and chemokine concentrations in feline whole blood. J Vet Emerg Crit Care (San Antonio).

[12] Heinz JA, Pashmakova MB, Wilson CR, Johnson MC, Minnard HM, Bishop MA, Barr JW (2016). Biochemical evaluation of the effects of storage on feline erythrocytes. J Small Anim Pract.

[13] Prittie JE (2010). Controversies related to red blood cell transfusion in critically ill patients. J Vet Emerg Crit Care (San Antonio).

[14] Bracker KE, Drellich S (2005). Transfusion reactions. Comp Cont Educ Pract Vet.

[15] Allen SE, Holm JL (2008). Lactate: physiology and clinical utility. J Vet Emerg Crit Care.

[16] Harvey JW, Weiss DJ, Wardrop KJ (2010). Erythrocyte biochemistry. Schalm’s veterinary hematology.

[17] Beutler E, Rossi EC, Simon TL, Moss GS (1996). Red cell metabolism. Principles of transfusion medicine.

[18] Rapoport I, Rapoport TA, Rapoport SM (1978). Analysis of pH-induced changes of the glycolysis of human erythrocytes. Acta Biol Med Ger.

[19] Beutler E, Rossi E, Simon T, Moss G (1996). Liquid preservation of red cells. Principles of transfusion medicine.

[20] Tavazzi B, Di Pierro D, Amorini AM, Fazzina G, Tuttobene M, Giardina B, Lazzarino G (2000). Energy metabolism and lipid peroxidation of human erythrocytes as a function of increased oxidative stress. Eur J Biochem.

[21] Picas L, Rico F, Deforet M, Scheuring S (2013). Structural and mechanical heterogeneity of the erythrocyte membrane reveals hallmarks of membrane stability. ACS Nano.

[22] Birka C, Lang PA, Kempe DS, Hoefling L, Tanneur V, Duranton C, Nammi S, Henke G, Myssina S, Krikov M, Huber SM, Wieder T, Lang F (2004). Enhanced susceptibility to erythrocyte “apoptosis” following phosphate depletion. Pflugers Arch.

[23] D'Alessandro A, Kriebardis AG, Rinalducci S, Antonelou MH, Hansen KC, Papassideri IS, Zolla L (2015). An update on red blood cell storage lesions, as gleaned through biochemistry and omics technologies. Transfusion.

[24] Mohandas N, Chasis JA (1993). Red blood cell deformability, membrane material properties and shape: regulation by transmembrane, skeletal and cytosolic proteins and lipids. Semin Hematol.

[25] Nakao M, Nakao T, Yamazoe S (1960). Adenosine triphosphate and maintenance of shape of the human red cells. Nature.

[26] Baskurt OK (1999). The role of spleen in suppressing the rheological alterations in circulating blood. Clin Hemorheol Microcirc.

[27] Greenwalt TJ, McGuinness CG, Dumaswala UJ (1991). Studies in red blood cell preservation: plasma vesicle hemoglobin exceeds free hemoglobin. Vox Sang.

[28] Högman CF, Meryman HT (2006). Red blood cells intended for transfusion: quality criteria revisited. Transfusion.

[29] Price GS, Armstrong PJ, McLeod DA, Babineau CA, Metcalf MR, Sellett LC (1988). Evaluation of citrate-phosphate-dextrose-adenine as a storage medium for packed canine erythrocytes. J Vet Intern Med.

[30] Wardrop KJ, Owen TJ, Meyers KM (1994). Evaluation of an additive solution for preservation of canine red blood cells. J Vet Intern Med.

[31] Kessler RJ, Rankin S, Young S, O’Shea K, Calabrese M, Guldin A, Lipson N, Oakley DA, Giger U (2010). Pseudomonas fluorescens contamination of a feline packed red blood cell unit and studies of canine units. Vet Clin Pathol.

[32] Sowemimo-Coker SO (2002). Red blood cell hemolysis during processing. Transfus Med Rev.

[33] Högman CF, Eriksson L, Ericson A, Reppucci AJ (1991). Storage of saline-adenine-glucose-mannitol-suspended red cells in a new plastic container: polyvinylchloride plasticized with butyryl-n-trihexyl-citrate. Transfusion.

[34] Boretti FS, Buehler PW, D'Agnillo F, Kluge K, Glaus T, Butt OI, Jia Y, Goede J, Pereira CP, Maggiorini M, Schoedon G, Alayash AI, Schaer DJ (2009). Sequestration of extracellular hemoglobin within a haptoglobin complex decreases its hypertensive and oxidative effects in dogs and Guinea pigs. J Clin Invest.

[35] Holland PV (1989). Standards for blood banks and transfusion services.

[36] Harrell KA, Kristensen AT (1995). Canine transfusion reactions and their management. Vet Clin North Am Small Anim Pract.

[37] Buehler PW, D'Agnillo F (2010). Toxicological consequences of extracellular hemoglobin: biochemical and physiological perspectives. Antioxid Redox Signal.

[38] Rother RP, Bell L, Hillmen P, Gladwin MT (2005). The clinical sequelae of intravascular hemolysis and extracellular plasma hemoglobin: a novel mechanism of human disease. JAMA.

[39] Amberson WR, Jennings JJ, Rhode CM (1949). Clinical experience with hemoglobin-saline solutions. J Appl Physiol.

[40] Savitsky JP, Doczi J, Black J, Arnold JD (1978). A clinical safety trial of stroma-free hemoglobin. Clin Pharmacol Ther.

[41] Deuel JW, Schaer CA, Boretti FS, Opitz L, Garcia-Rubio I, Baek JH, Spahn DR, Buehler PW, Schaer DJ (2016). Hemoglobinuria-related acute kidney injury is driven by intrarenal oxidative reactions triggering a heme toxicity response. Cell Death Dis.

[42] Harrison HE, Bunting H, Ordway NK, Albrink WS (1947). The pathogenesis of the renal injury produced in the dog by hemoglobin or methemoglobin. J Exp Med.

[43] Schechter AN, Gladwin MT (2003). Hemoglobin and the paracrine and endocrine functions of nitric oxide. N Engl J Med.

[44] Roback JD (2011). Vascular effects of the red cell storage lesion hematology. Am Soc Hematol Educ Program.

[45] Burhop K, Gordon D, Estep T (2004). Review of hemoglobin-induced myocardial lesions. Artif Cells Blood Substit Immobil Biotechnol.

[46] Koch CG, Li L, Sessler DI, Figueroa P, Hoeltge GA, Mihaljevic T, Blackstone EH (2008). Duration of red-cell storage and complications after cardiac surgery. N Engl J Med.

[47] Council of Europe (2015). Component monographs part B. Red cell components. Guide to the preparation, use and quality assurance of blood components.

[48] Kakaiya R, Colleen AA, Julleis J, Brecher ME (2012). Whole blood collection and component processing at blood collection centers. American association of blood banks technical manual.

[49] Barfield D, Adamantos S (2011). Feline blood transfusions: a pinker shade of pale. J Feline Med Surg.

[50] Spada E, Proverbio D, Bagnagatti De Giorgi G, Perego R, Valena E, Della Pepa A (2015). Clinical and haematological responses of feline blood donors anaesthetised with a tiletamine and zolazepam combination. J. Feline Med. Surg.

[51] Feldman BF, Sink CA, Feldman BF, Sink CA (2008). Collection, processing, storage and shipment. Practical transfusion medicine for the small animal practitioner.

[52] Brown BA, Brown BA (1984). Hematology: principles and procedures. Routine hematology procedures.

[53] Sawant RB, Jathar SK, Rajadhyaksha SB, Kadam PT (2007). Red cell hemolysis during processing and storage. Asian J Transfus Sci.

[54] Janatpour KA, Paglieroni TG, Crocker VL, DuBois DJ, Holland PV (2004). Visual assessment of hemolysis in red blood cell units and segments can be deceptive. Transfusion.

[55] Zimmermann R, Heidenreich D, Weisbach V, Zingsem J, Neidhardt B, Eckstein R (2003). In vitro quality control of red blood cell concentrates outdated in clinical practice. Transfus Clin Biol.

[56] Zehnder L, Schulzki T, Goede JS, Hayes J, Reinhart WH (2008). Erythrocyte storage in hypertonic (SAGM) or isotonic (PAGGSM) conservation medium: influence on cell properties. Vox Sang.

[57] Carter MW, Matrone G, Mendenhall W (1964). Estimation of the life span of red blood cells. J Gen Physiol.

[58] Ferreira RR, Graça R, Cardoso IM, Ruiz de Gopegui R, De Matos AJ. (in press)In vitro hemolysis during storage of canine packed red blood cells. J Vet Emerg Crit Care. 10.1111/vec.12770 30299571

[59] Jain NC (1973). Osmotic fragility of erythrocytes of dogs and cats in health and in certain hematologic disorders. Cornell Vet.

[60] Hohenhaus AE, Drusin LM, Garvey MS (1997). Serratia marcescens contamination of feline whole blood in a hospital blood bank. J Am Vet Med Assoc.

[61] Stefanetti V, Miglio A, Cappelli K, Capomaccio S, Sgariglia E, Marenzoni ML, Antognoni MT, Coletti M, Mangili V, Passamonti F (2016). Detection of bacterial contamination and DNA quantification in stored blood units in 2 veterinary hospitalblood banks. Vet Clin Pathol.

[62] Spada E, Proverbio D, Martino PA, Perego R, Baggianil L, Roggero N. Ammonia concentration and bacterial evaluation of feline whole blood and packed red blood cell units stored for transfusion. Int J of Health, Animal Sci Food Safety, Vol. 1. 2014:15–23.

[63] Mansell CL, Boller M, Yagi K, Holowaychuk MK (2016). Blood comoponent processing and storage. Manual of veterinary transfusion medicine and blood banking.

